# Preliminary application of Apple Vision Pro (AVP) in laparoscopic gastrointestinal surgery

**DOI:** 10.1097/JS9.0000000000003066

**Published:** 2025-07-17

**Authors:** Kexin Shen, Wenhui Li, Zhongshi Xie

**Affiliations:** a Department of Gastrointestinal Colorectal and Anal Surgery, China-Japan Union Hospital of Jilin University, Changchun, Jilin, P. R. China; b Department of Ultrasound, China-Japan Union Hospital of Jilin University, Changchun, Jilin, P. R. China

**Keywords:** Apple Vision Pro, colorectal cancer, laparoscopic surgery

## Abstract

**Objective:**

The aim of this article is to investigate the application of Apple Vision Pro (AVP) in laparoscopic gastrointestinal surgery.

**Methods:**

In this study, AVP was used for the first time in a delicate medical procedure such as laparoscopic abdominal and gastrointestinal surgery. A total of 22 cases were included, involving malignant tumors of the ascending colon, sigmoid colon, and rectum. The safety of AVP in laparoscopic surgery was evaluated from three perspectives: surgical safety, information security, and physician safety.

**Result:**

All the patients successfully completed the operation, and the video signal was transmitted smoothly during the operation. The average operation time was 189 min. All patients recovered smoothly after the operation, and no obvious complications occurred. The surgeon had no discomfort with wearing AVP.

**Conclusion:**

AVP is safe and effective in laparoscopic gastrointestinal surgery, although some limitations were noted. AVP shows great potential in medical applications, education, and hospital equipment. We envision that AVP will significantly support the advancement of laparoscopic surgery, providing surgeons with a more precise, efficient, and safer surgical environment, thereby promoting progress in healthcare.

## Introduction

Since the early 1990s, laparoscopic surgery has revolutionized general surgery^[1]^ and become a standard method for abdominal minimally invasive procedures^[2]^. Despite the gradual improvements in display resolution and size in the field of endoscopic surgery, the relatively fixed position and dimensions of the displays continue to impose a significant burden on surgeons^[3]^. This prompts the search for better solutions.

Augmented reality (AR) technology is a new practical technology developed in the 20th century. AR technology takes the physical information that is difficult to experience in the real-world space, such as visual information, sound, and taste, superimposes it to the real world through scientific and technological simulation, and is perceived by the user’s senses so as to achieve a sensory experience beyond reality^[4]^. At present, AR technology has been applied in many fields such as network communication, television broadcasting, and electronic games and has achieved surprising results and shown great application prospects^[5]^. AR technology also has great application potential in the medical field, and at present, doctors have applied AR technology to the diagnosis and treatment of diseases^[6,7]^. June 2023 Apple releases its first “Spatial Computing” device, the Apple Vision Pro (AVP)^[8]^. It is mixed reality (MR) glasses with AR and virtual reality (VR) functions, equipped with 12 sensors, five cameras, and six microphones, which can be controlled by the user through gestures, eyes, or words. It is a new generation of electronic products that can be used for work, entertainment, and communication. Regarding the possibility of application of AVP in the medical field, there are controversial^[9]^ and related application cases^[10–13]^. This study applied AVP to laparoscopic surgery in gastrointestinal surgery for the first time and discussed the feasibility of the application of this new technology and new equipment in laparoscopic surgery. This study has been reported in accordance with the TITAN criteria, which are cited in the reference list^[14]^. This case series has been reported in line with the PROCESS guidelines^[15]^.

**HIGHLIGHTS**

- The Apple Vision Pro headset was first used in laparoscopic surgery.
- Multi-screen linkage in the real world with AR technology has replaced the traditional screen.
- The modularity of laparoscopic colorectal surgery facilitates learning.
- The strategic placement of display windows helps to alleviate surgeon fatigue.

## Materials and methods

The article has been checked against the SQUIRE checklist^[16]^.

### Patients and surgeon

A total of 22 patients with colorectal cancer who visited our department at different stages of disease progression from May to August 2024 were continuously included (Table 1). The surgical procedure was explained in detail to the patients before surgery, and all patients signed informed consent. All 22 cases in this study were completed by a surgical team led by the operator, who has been engaged in gastrointestinal surgery for 15 years and has rich clinical experience. The operator wore AVP throughout the operation and evaluated the application of AVP in gastrointestinal surgery after the operation.

**Table 1:** Patients’ information

Basic information						Concomitant disease		Operation protocol				Complications	Postoperative pathology		
No.	Sex	Age (years)	BMI	Tumor location	Distance to anus	Hypertension	Diabetes	Operation time (m)	Lymph node dissection	Neostomy	Blood loss (mL)		Gross type	Diameter (cm)	Staging
1	F	53	27.48	Rectum	Medium (6–12 cm)	None	None	119	D3	None	10.6	-	Mass	3.8	I
2	F	64	23.24	Ascending colon		None	None	115	D3	None	42.5	-	Ulcerative	6	II
3	M	77	22.15	Sigmoid colon	High (>12 cm)	None	None	59	D3	None	15.6	-	Mass	2.3	I
4	M	72	17.63	Rectum	Medium (6–12 cm)	High risk	None	126	D3	Ileostomy	10.6	-	Mass	3.4	II
5	F	67	22.48	Ascending colon	High (>12 cm)	None	None	136	D3	None	43.6	-	Ulcerative	6	III
6	M	56	21.22	Rectum	Medium (6–12 cm)	None	None	191	D3	Ileostomy	26.6	-	Ulcerative	5.5	II
7	M	63	25.25	Rectum	Medium (6–12 cm)	None	None	299	D3	Ileostomy	24.5	-	Mass	6	II
8	F	67	24.91	Rectum	Medium (6–12 cm)	High risk	None	275	D1	Ileostomy	41.7	-	Infiltrate	3.5	III
9	M	61	26	Rectum	Medium (6–12 cm)	None	None	281	D3	None	11.3	-	Mass	2.4	III
10	F	75	24.4	Sigmoid colon	High (>12 cm)	High risk	None	204	D3	None	24.3	-	Ulcerative	5	III
11	M	53	24.4	Rectum	Medium (6–12 cm)	None	None	209	D3	None	26.8	-	Ulcerative	5.5	II
12	M	61	21.72	Rectum	Medium (6–12 cm)	Low risk	None	93	D3	None	10.4	-	Ulcerative	5.5	III
13	F	42	31.22	Rectum	Medium (6–12 cm)	None	None	180	D3	Ileostomy	57.9	-	Ulcerative	4	III
14	M	59	24.81	Rectum	Low (<6 cm)	None	None	291	D3	Ileostomy	33.4	-	Ulcerative	Unknown	I
15	M	71	25.69	Sigmoid colon	High (>12 cm)	High risk	None	158	D3	Ileostomy	19.4	-	Ulcerative	5	III
16	M	70	23.01	Rectum	Medium (6–12 cm)	None	None	172	D3	None	16.7	-	Mass	Unknown	III
17	M	74	17.63	Rectum	Medium (6–12 cm)	None	None	198	D3	Ileostomy	12.4	-	Ulcerative	4	II
18	M	52	26.44	Rectum	Low (<6 cm)	None	Type 2	295	D3	Ileostomy	13.6	-	Mass	4.8	II
19	M	78	27.34	Rectum	High (>12 cm)	None	None	229	D3	Ileostomy	12.4	-	Infiltrate	3.5	IV
20	M	50	21.47	Sigmoid colon	High (>12 cm)	None	None	141	D3	None	7.4	-	Ulcerative	4.2	II
21	F	49	24.22	Rectum	Medium (6–12 cm)	None	None	239	D3	Ileostomy	24.7	-	Ulcerative	5	II
22	M	56	23.3	Rectum	Low (<6 cm)	None	None	150	D3	Ileostomy	71.9	-	Ulcerative	3.3	II

### Preoperative preparation

#### Equipment preparation

The operator wears an AVP (The Apple Vision Pro, Apple Inc., Cupertino, CA, USA) before brushing his hands and putting on a sterile surgical gown.

Prior to surgery, the video signal is connected by the following process:

1. Acquisition card connection: Connect the HAGiBiS acquisition card to the display output end of the laparoscope, and connect the output end of the acquisition card to the MacBook through the USB-C interface, so that the MacBook can obtain images of the laparoscopic camera in real time.
2. Capture software configuration: Run the OBS capture software on the MacBook to adjust the resolution to 960 × 740, reduce the video processing capacity, and thus reduce the delay time. In the initial state, the delay with the Hypersis capture card is about 120 ms, but the delay can be optimized to less than 100 ms by adjusting the resolution to 960 × 740.

#### Patient data preparation

Patients’ basic data (gender, age, height, weight, and admission diagnosis) were recorded before surgery. Meanwhile, preoperative examination results (colonoscopy, pathology, and tumor location images shown by CT, MRI, and ultrasound) were also imported into AVP after hiding patients’ private information.

### Surgical procedure

**Figure 1.**
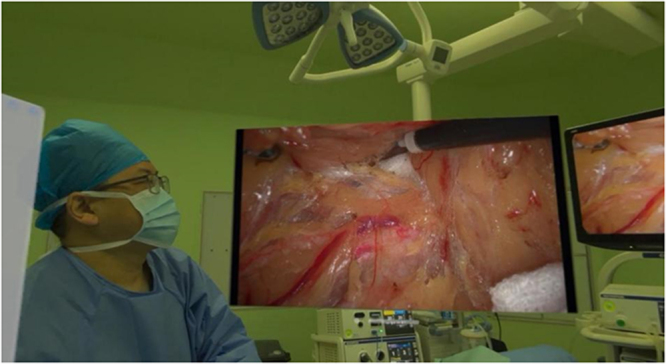
The Apple Vision Pro offers a larger display window in high definition. On the right is the display size of the original laparoscopic device.

**Figure 2.**
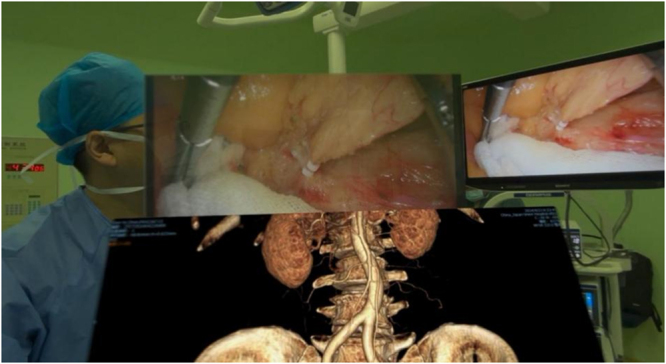
The Apple Vision Pro’s multi-window display enables real-time navigation of vascular anatomy during surgery.

When surgeons wear AVP, they can obtain a clearer and larger display window in real time than traditional laparoscopic display devices (Fig. 1). When necessary, patients’ clinical data can be viewed through multiple Windows, and the intraoperative vascular anatomy can be navigated in real time to provide guidance for the location and routing of key blood vessels (Fig. 2). The laparoscope is equipped with a fixed display for assistant use.

### Questionnaire

**Figure 3.**
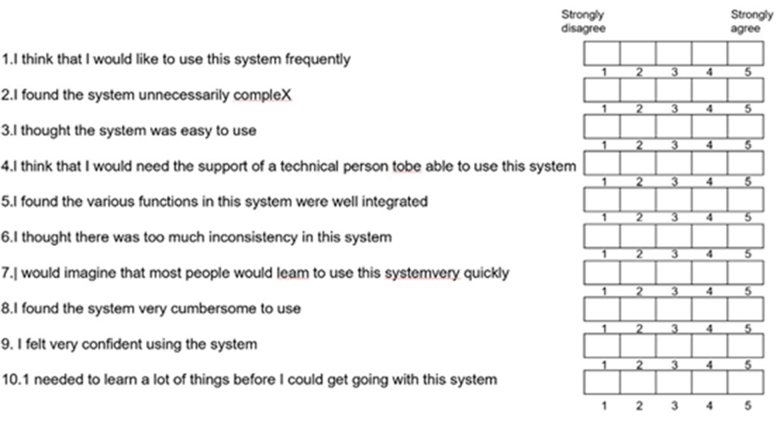
System Usability Scale (SUS).

The operator, the AVP wearer, completed the validated usability questionnaire, System Usability Scale (SUS), after each surgery to evaluate the feasibility of applying AVP in procedures of varying difficulty levels (Fig. 3). The SUS consists of 10 items (statements) scored on a 5-point Likert scale (1 = strongly disagree to 5 = strongly agree). It measures product usability across three different dimensions: effectiveness, efficiency, and satisfaction. The scores of each questionnaire were calculated and analyzed. The SUS score is calculated by first determining the base value for each question, which ranges from 0 to 4. The base value is computed as follows:

- For odd-numbered items, subtract 1 from the score (e.g., if the score for Question 1 is 3, the base value is 3 − 1 = 2).
- For even-numbered items, subtract the score from 5 (e.g., if the score for Question 2 is 3, the base value is 5 − 3 = 2).

The SUS score is then obtained by summing the base values of all 10 questions and multiplying the total by 2.5 (since each question’s base value ranges from 0 to 4, and the SUS usability score ranges from 0 to 100, the conversion requires multiplying by 2.5). Descriptive statistics are presented as mean ± standard deviation and median, with calculations performed using SPSS 27.0 software (SPSS Inc., Chicago, IL, USA).

## Results

### Patients

This study included 22 cases: 15 cases (68.18%) were male and 7 cases (31.82%) were female. The mean age was 62.27 ± 10.119 years. There were 15 rectal malignancies (12 median, 3 low), 4 sigmoid malignancies (all high), 2 ascending colon malignancies, and 1 descending colon malignancies. The operation time was 59–299 min (189.09 ± 69.978), of which 9 patients (40.91%) took more than 200 min, 5 patients (22.73%) took more than 240 min, and 12 patients (54.55%) took preventive ostomy. Intraoperative blood loss was 7.4–71.9 mL (25.377 ± 16.9714), and no postoperative complications occurred (Table 1). Based on surgical difficulty, the average SUS scores from most difficult to easiest were as follows: ascending colon tumor (90 points), low rectal tumor (90 points), mid-rectal tumor (90 points), high-risk descending colon tumor (95 points), and sigmoid colon tumor (100 points).

### Video signal and user experience

The video signal transfer and debugging process takes 10 min, and the video display resolution is 4K. The frequency of video signal interruption and blurring during the whole operation was 0. The initial latency of the video signal was 120 ms when the MacBook was connected using a Hypersis capture card. When the resolution of the OBS capture software is set to 960 × 740, the video latency is reduced to less than 100 ms. Upgrade to an Elgato 4K capture card and Mac Studio computer with 1080p resolution and reduced latency to 60 ms. During the operation, the assistant and the instrument nurse can see the surgeon’s eyes through the lens, and the communication efficiency between them is not affected. According to the needs of the operation, the surgeon can access the preoperative examination data and other data through the contactless adjustment of the Digital Crown button of the AVP. In this study, when three surgeries were performed, the maximum daily wearing time was 7.41 h, and the device could still maintain clear image display and comfortable wearing experience after wearing.

## Discussion

This study shows that AVP is safe and effective in laparoscopic gastrointestinal surgery. Since the release of AVP, researchers and doctors from all over the world have looked forward to the application of AVP in medicine, and they all believe that AVP will shine in the medical field and promote the development of precision medicine^[4,17]^. However, the actual clinical application of AVP was limited to ophthalmology^[18]^, dermatology^[10]^, plastic surgery^[19]^, and neurosurgery^[20]^, and there were no reports on the application of AVP to laparoscopic surgery in gastrointestinal surgery. This study shared 22 cases of laparoscopic surgery related to gastrointestinal surgery performed with AVP to evaluate the application value of AVP in gastrointestinal surgery.

**Figure 4.**
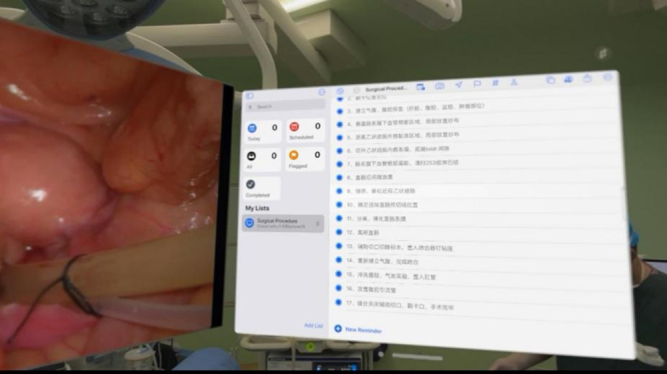
The Apple Vision Pro records surgical procedures in real time to standardize the surgical process.

**Figure 5.**
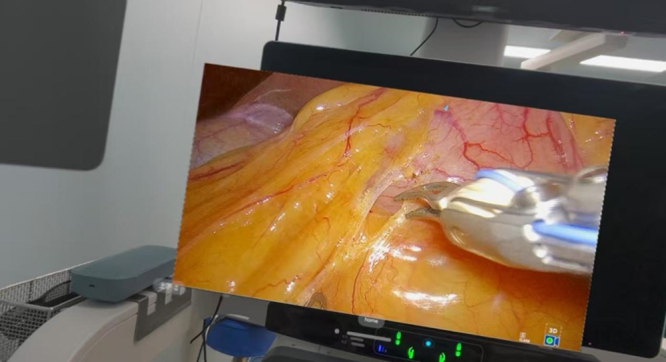
AVP’s ultra-high resolution display system has the same clarity as traditional laparoscopic displays.

We found that the application of AVP in laparoscopic gastrointestinal surgery was satisfactory in terms of operation safety, information security, and physician safety. First of all, all the operations were successfully completed, and the average operation time, intraoperative blood loss, and complication rate of the 22 cases included in this study were not different from the previous laparoscopic surgery data of our team without AVP. During the operation, there was no interruption or blurring of the video signal that affected the operation process. The cases included in this study included malignant tumors of ascending colon, sigmoid colon, and rectum, and 55.55% of the cases underwent preventive ostomy. The cases in this group covered different surgical difficulties, all of which were delicate operations, requiring high accuracy and reliability of image support. All of the surgical procedures in this study went smoothly (Fig. 4), starting with the ultra-high resolution display system relying on AVP, which uses microOLED technology to pack 23 million pixels on two displays, and the single-eye pixels exceed 4K display, with no difference in clarity from traditional laparoscopic displays (Fig. 5). Second, AVP’s high-performance tracking system uses a high-speed camera and ring LED to project invisible light patterns on the user’s eyes to achieve rapid response and intuitive eye tracking, and is equipped with a high-speed camera to capture hand movements. In this study, the delay time of the surgical video transmission signal is 60 ms, which is almost no perceptible delay to the naked eye. Finally, there is the dual-chip design of the AVP, powered by the unique M2+R1 dual-chip design of the Apple networking chip. The M2 chip provides excellent independent performance, and the new R1 chip processes inputs from 12 cameras, 5 sensors, and 6 microphones and can generate a low-latency display stream within 12 ms to ensure the fluency of the picture^[8]^ and ensure the smooth transmission of surgical video. In this study, there was no impact on the coordination between the surgeon and the assistant.

In this study, the team members collated the patient data in advance, hid the basic information, and then transmitted it to the AVP. During the operation, the role of the AVP was to transmit the laparoscopic video signal to the device without any network data transmission. In addition, AVP adopts iris recognition technology^[4]^, only the surgeon, the operator, can wear and access the patient data stored in AVP, and there is no leakage of patient privacy information. The key point of information security provides the possibility for the application of AVP in public medical institutions.

**Figure 6.**
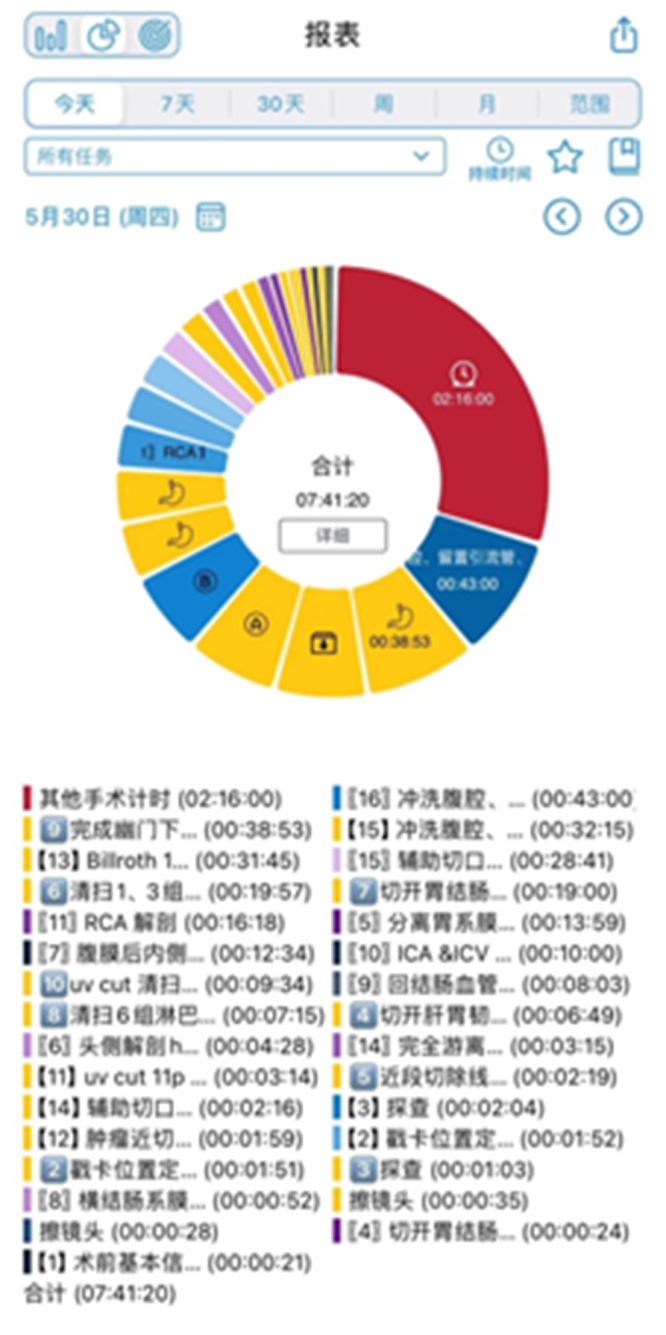
The AVP devices in this study were worn for up to 7 h, 41 min, and 20 s.

Some studies have shown that wearing a head-mounted VR display for long periods of time can disrupt the sensory system, causing symptoms such as nausea, dizziness, sweating, paleness, and loss of balance, which are collectively referred to as “VR disease”^[21]^. For sensitive people, these symptoms may appear within minutes of using VR. AR/VR devices use LED screens that may contain a lot of blue light, which can disrupt biorhythms (delayed sleep patterns, disturbed sleep, etc.) when used at night. The AVP device in this study was worn by the operator for a maximum of 7 h, 41 min, and 20 s (Fig. 6). According to his feedback, he had worn AVP for surgery for 6 months, and his vision and sensory system were not affected, and he did not experience the related symptoms of VR disease mentioned earlier. Only the weight of the AVP caused the forehead skin indentation, the pressure on the head and neck was small, and the display clarity and stability of the device remained good during use. In Orione *et al*’s study^[22]^, consistent with this study, only external discomfort caused by AVP self-weight was reported, and the appearance of VR syndrome was not mentioned. Therefore, this study concluded that surgeons wearing AVP for a long period of surgery will not affect their health.

**Figure 7.**
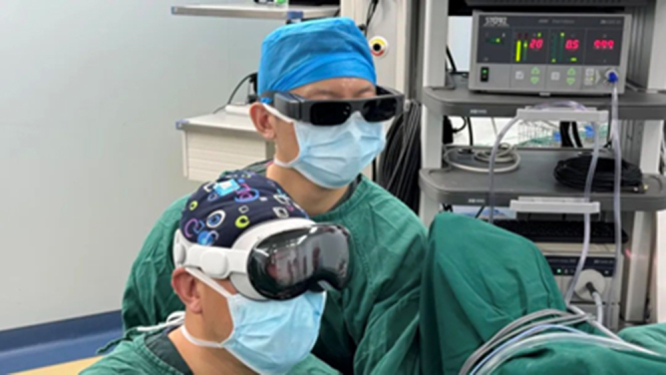
During the abdominal and anal procedure, the two surgeons can each watch a screen.

**Figure 8.**
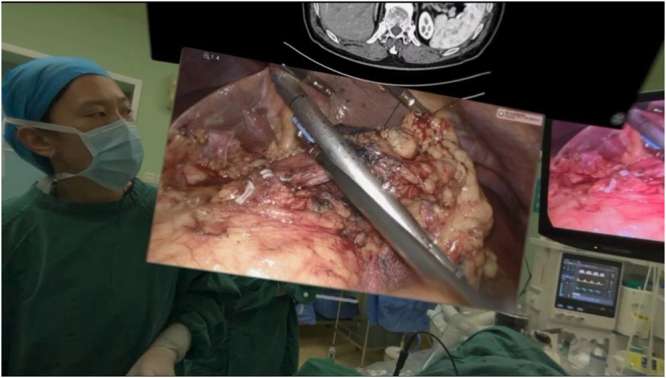
In patients with hypertrophic mesangial tissue, CT examination images during the operation are more conducive to tumor location.

Our research also demonstrates that using AVP can improve daily productivity. First of all, AVP is equivalent to adding a mobile display, the first assistant can directly see the image of the surgical area through the device, no longer need to side head to view the external display, significantly alleviating the problem of cervical fatigue. Traditional laparoscopic surgery often uses fixed-position displays to display surgical operation scenes. However, due to the limitations of the optical block resolution of laparoscopic equipment and the size of the display, it may not be able to provide high-definition images satisfactory enough to surgeons. When the surgical area is transformed, the viewing angle of the surgeon and his assistant is often limited. More uncomfortable positions, such as twisting the neck, are needed to view the monitor effectively. Limited hardware facilities to a certain extent affect the surgeon’s observation of the surgical area and the accuracy of operation. Uncomfortable posture accelerates the fatigue of the surgeon and his assistant, which not only affects the efficiency of surgery but also increases the risk of surgery^[4,23]^. The AVP puts a high-definition display window in front of the surgeon without twisting the body to look at the display. For example, in gastric cancer surgery, the high-quality image provided by the AVP helps to more accurately locate the lesion and perform the operation. Especially in the detailed operation, the visual fluency provided by the head-mounted display greatly improves efficiency and safety. Second, AVP enables the surgical team to operate at multiple angles without interference. During transanal total mesorectal resection, AVP can display different viewing angles during transabdominal and transanal operation, and the two surgeons can each see a “screen” (Fig. 7). The surgeon can also adjust the size of the display window at will, which can fully enlarge the surgical area and observe the anatomical details of the tissue, improving the comfort of the surgeon. Reducing the mental load of the surgeon and avoiding disorientation can help the surgeon concentrate on the surgical process and improve the control of the subtle operation. In addition, AVP enables real-time data display and real-time navigation and guidance during surgery. AVP can not only set the display window at will in the real three-dimensional space, but also connect with the patient’s clinical data through the surgeon’s own access or remote delivery by the assistant, and display the surgical area and patient-related data through multiple Windows. In case 13 of this study, the mesangial tissue of the patient was relatively hypertrophic, but the tumor volume was relatively small. Real-time CT inspection during the operation is more conducive to tumor location (Fig. 8). In the treatment of mesangial blood vessels, the positioning and running information of the target blood vessels of the patient can be displayed in real time, guiding the direction of tissue free, assisting the surgical operation, making the surgical process more accurate, and improving the surgical efficiency. Finally, another important role of AVP is the normalization of surgical procedures. For the same type of surgery, setting standardized operating procedures in advance and recording operational steps in real time are more conducive to the surgical personnel’s grasp of the surgical process and rhythm, and can check the shortcomings and make up for the omissions in real time with reference to the surgical process, which can not only improve the standardization and homogenization of the surgery but also avoid some unnecessary negligence, reduce the risk of surgery, and improve the efficiency of surgery.

The application of AVP in laparoscopic surgery in this study is only a new beginning. In this era of the “Internet of everything,” AVP has great potential applications, first in the field of medicine^[24]^, preoperative diagnostic assistance, intraoperative improvement of surgical procedures, remote care of patients at any time after surgery, and evaluation of medical records. In the field of medical education^[25]^, AVP can promote the “modularization” of surgery, such as the completion of corresponding necessary operations in a certain surgical area, which is conducive to the learning and understanding of surgery for young physicians. In the future, AVP is expected to be further developed to realize that through AVP, surgical operation can be simulated anytime and anywhere, promoting the development of simulated surgical teaching, and even combining real patient data, clinicians can perform simulated surgical rehearsal before real surgery, which will greatly improve surgical safety. Finally, in terms of medical equipment, laparoscopic surgery involves more equipment, and the space around the operating bed is limited. Traditional laparoscopic equipment and fixed displays need to occupy a certain space. For the operating room with insufficient area, the operating room activity space will be limited. In order to improve the clarity of the display, some laparoscopic devices increase the size of the display, further squeezing the limited space around the operating bed, and thus increasing the inconvenience. In addition, adjustments to the equipment or display may affect the surgical process and the operation of the surgical personnel. The AVP can place the display window at will in the realistic three-dimensional space to realize the “de-display” of laparoscopic surgery and save limited space. In the future, if the AVP can be connected with the Da Vinci Robot surgical system, it may be possible to further reduce the size of the doctor’s console and potentially reduce the cost and save limited medical resources.

However, our study found that there are still some shortcomings in the application of AVP in laparoscopic surgery, the first is the weight of the equipment, the surgical operation usually takes more than 2 h, although the device is relatively lightweight design, it may still cause certain pressure on the head, especially after continuous use for several hours, the comfort of the head and neck is slightly affected. The second problem is battery life: the AVP built-in battery has limited battery life in high-definition display mode. It is recommended to connect an external power source during a long procedure to ensure continuous high-quality image support throughout the procedure. At the same time, in the case of low power, the system will automatically reduce the resolution to extend the use time, but this will affect the fineness of the image, affecting the visual needs in complex operations. In addition, there is the preparation process required for video signal transmission, which needs to go through multi-party switching, which is more complicated, and there is the situation of electrical equipment interfering with the video signal, which needs to be improved. Finally, it is expensive and difficult to popularize at present, affecting its wide application in the medical field. These findings are consistent with the results reported by Armstrong *et al*^[9]^.

## Conclusion

Our study proves that AVP laparoscopic surgery shows reliable safety and high effectiveness, bringing new changes to laparoscopic surgery, and is expected to provide important support for the development and progress of minimally invasive surgery technology, provide doctors with a more accurate, efficient, and safe surgical environment, and promote the development and progress of medical care.

## Data Availability

Data sharing is not applicable to this article.

## Research registration unique identifying number (UIN)

This article does not involve human research.

## Publication history

This manuscript has never been published anywhere.
